# Interpretation of Thoracic Radiography Shows Large Discrepancies Depending on the Qualification of the Physician—Quantitative Evaluation of Interobserver Agreement in a Representative Emergency Department Scenario

**DOI:** 10.3390/diagnostics11101868

**Published:** 2021-10-11

**Authors:** Jan Rudolph, Nicola Fink, Julien Dinkel, Vanessa Koliogiannis, Vincent Schwarze, Sophia Goller, Bernd Erber, Thomas Geyer, Boj Friedrich Hoppe, Maximilian Fischer, Najib Ben Khaled, Maximilian Jörgens, Jens Ricke, Johannes Rueckel, Bastian Oliver Sabel

**Affiliations:** 1 Department of Radiology, University Hospital, LMU Munich, Marchioninistr. 15, 81377 Munich, Germany; nicola.fink@med.uni-muenchen.de (N.F.); julien.dinkel@med.uni-muenchen.de (J.D.); vanessa.koliogiannis@med.uni-muenchen.de (V.K.); vincent.schwarze@med.uni-muenchen.de (V.S.); sophia.goller@med.uni-muenchen.de (S.G.); bernd.erber@med.uni-muenchen.de (B.E.); thomas.geyer@med.uni-muenchen.de (T.G.); boj.hoppe@med.uni-muenchen.de (B.F.H.); jens.ricke@med.uni-muenchen.de (J.R.); johannes.rueckel@med.uni-muenchen.de (J.R.); bastian.sabel@med.uni-muenchen.de (B.O.S.); 2 Comprehensive Pneumology Center (CPC-M), German Center for Lung Research, Max-Lebsche-Platz 31, 81377 Munich, Germany; 3 Department of Radiology, Asklepios Fachklinik München, Robert-Koch-Allee 2, 82131 Gauting, Germany; 4 Department of Medicine I, University Hospital, LMU Munich, Marchioninistr. 15, 81377 Munich, Germany; maximilian.fischer@med.uni-muenchen.de; 5 Department of Medicine II, University Hospital, LMU Munich, Marchioninistr. 15, 81377 Munich, Germany; najib.benkhaled@med.uni-muenchen.de; 6 Department of Orthopaedics and Trauma Surgery, Musculoskeletal University Center Munich (MUM), University Hospital, LMU Munich, Marchioninistr. 15, 81377 Munich, Germany; maximilian.joergens@med.uni-muenchen.de

**Keywords:** chest radiography, emergency department, interrater reliability, radiologists, clinicians

## Abstract

(1) Background: Chest radiography (CXR) is still a key diagnostic component in the emergency department (ED). Correct interpretation is essential since some pathologies require urgent treatment. This study quantifies potential discrepancies in CXR analysis between radiologists and non-radiology physicians in training with ED experience. (2) Methods: Nine differently qualified physicians (three board-certified radiologists [BCR], three radiology residents [RR], and three non-radiology residents involved in ED [NRR]) evaluated a series of 563 posterior-anterior CXR images by quantifying suspicion for four relevant pathologies: pleural effusion, pneumothorax, pneumonia, and pulmonary nodules. Reading results were noted separately for each hemithorax on a Likert scale (0–4; 0: no suspicion of pathology, 4: safe existence of pathology) adding up to a total of 40,536 reported pathology suspicions. Interrater reliability/correlation and Kruskal–Wallis tests were performed for statistical analysis. (3) Results: While interrater reliability was good among radiologists, major discrepancies between radiologists’ and non-radiologists’ reading results could be observed in all pathologies. Highest overall interrater agreement was found for pneumothorax detection and lowest agreement in raising suspicion for malignancy suspicious nodules. Pleural effusion and pneumonia were often suspected with indifferent choices (1–3). In terms of pneumothorax detection, all readers mainly decided for a clear option (0 or 4). Interrater reliability was usually higher when evaluating the right hemithorax (all pathologies except pneumothorax). (4) Conclusions: Quantified CXR interrater reliability analysis displays a general uncertainty and strongly depends on medical training. NRR can benefit from radiology reporting in terms of time efficiency and diagnostic accuracy. CXR evaluation of long-time trained ED specialists has not been tested.

## 1. Introduction

Chest radiography (CXR) still represents one of the most commonly required examinations in emergency departments (ED) and makes up a key component in primary diagnostics [1,2,3,4,5]. In our clinic’s emergency department, we performed a total of 4081 chest radiographs (CXRs) in 2020 (5351 CXRs in 2019—smaller numbers in 2020 might be explained by an overall decrease of patient presentations in ED due to the COVID-19 pandemic).

Typical findings in CXR include consolidations suspicious of pneumonia, pleural effusions, pneumothorax and pulmonary nodules. With estimated and/or approximated incidences of 1.5 to 14.0 (pneumonia, [6]), up to 322.7 (pleural effusion, [7]), 22.7 (pneumothorax, [8]) and 6.6 to 12.6 per 100,000 patients per year (pulmonary nodules, [9]), all mentioned diseases occur very frequently. Ideally, all of them should be diagnosed at early stages as their occurrence might require an urgent follow-up intervention (e.g., insertion of a thoracic tube in an extensive pneumothorax or pleural effusion) or patients can strongly benefit from an immediate therapy (e.g., bacterial/fungal pneumonia, pulmonary nodules). In addition, in pleural effusions, the appearance may provide an indication of the underlying primary disease (e.g., cardiac decompensation, malignancy).

Over the years, a number of studies has shown that correct interpretation of CXRs can be a major difficulty for radiologists as well as for clinicians due to low sensitivity for most of the common findings [1,10,11,12,13,14]. In the considered scenario of the emergency unit radiologists as well as non-radiological clinicians are confronted with CXR reporting. Often very young physicians in training (radiologists and non-radiologists) are the first diagnosticians to interpret the images, therefore having the responsibility to identify several urgent pathologies and draw consequences. In a setting without 24/7 coverage of a radiology department (e.g., in smaller hospitals), reporting might be even performed exclusively by non-radiologists, frequently being very young clinicians in training. To date, no study has specifically looked at a representative CXR imaging dataset from the emergency department in order to compare radiologists’ and non-radiologists’ image interpretation.

In this context, the present work aims to quantify interobserver agreement in CXR diagnostics taking place in emergency departments and to identify potential discrepancies that occur between different groups of CXR readers (board-certified radiologists, radiology residents, and non-radiology residents).

## 2. Materials and Methods

The study has been approved by the institutional ethics committee (approval number 19-0541) and federal authorities (General Administration of the Free State of Bavaria).

### 2.1. Patient Identification and Reading

**Figure 1:**
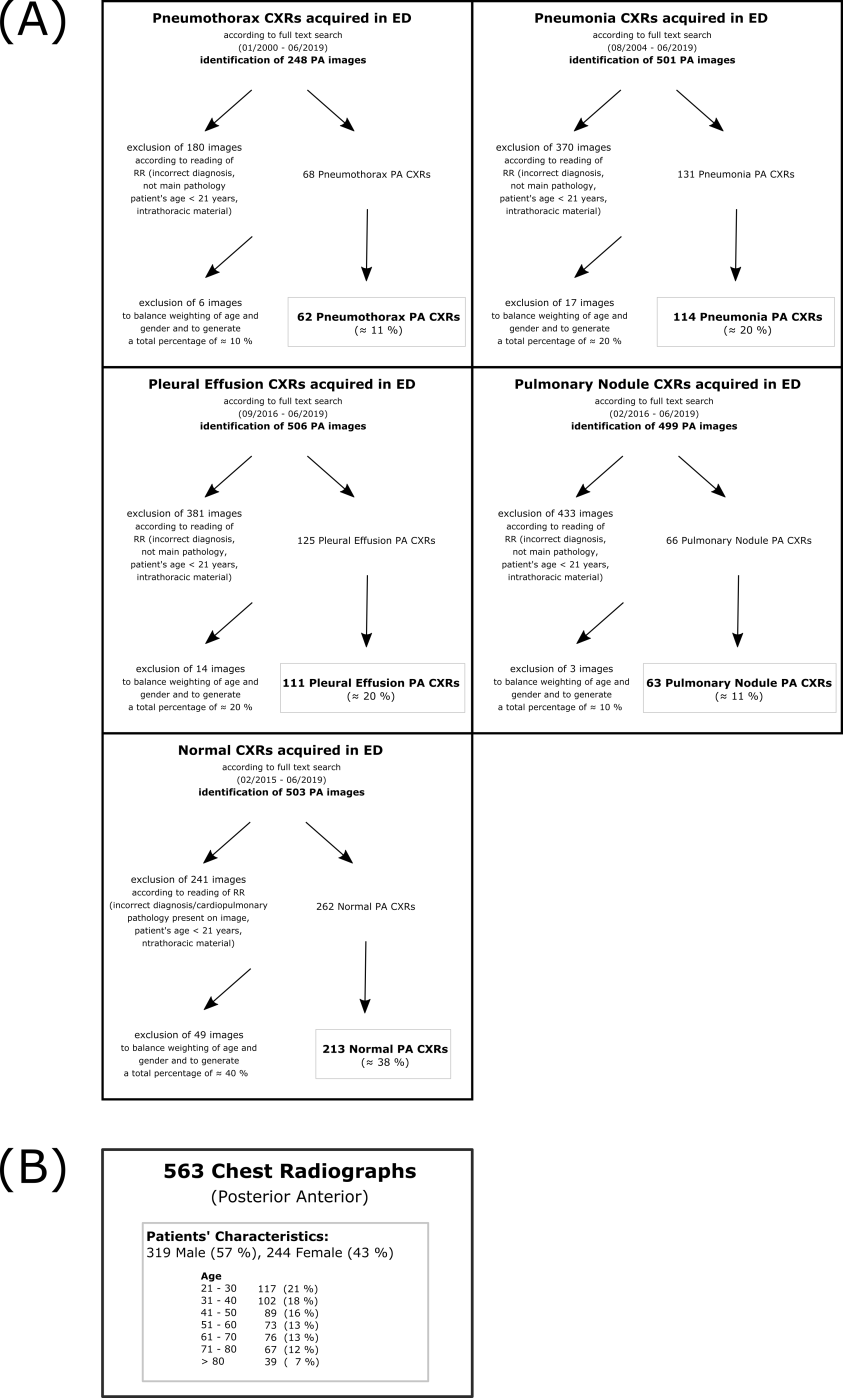
Preselection of study cohort—(**A**) Flow charts that display the preselection process of each subcohort (normal, pneumothorax, pneumonia, pleural effusion and pulmonary nodule). Images were identified by full text search in the local PACS. All images were preread by a radiology resident not participating in the main reading process. Images that did not meet inclusion criteria (correct diagnosis, main pathology, patient’s age ≥ 21, no foreign material) were excluded. After a first preselection, further random images were excluded to balance out quantities in terms of age and gender in the different cohorts; (**B**) shows the overall patient’s characteristics in the final cohort. Notice that the preselection was based on the main pathology which means that also more than one pathology was possible (e.g., pleural effusion + basal consolidation or pneumothorax + pleural effusion). Frequencies could therefore also differ from board-certified radiologists’ evaluation (see Figure 2).

CXR images were retrospectively identified by a full text data research in the institutional Picture Archiving and Communication System (PACS); search criteria were based on radiology reports from 2000–2019. Recruitment criteria were: patient presentation at the emergency unit attached to the local university clinic, patient’s age ≥ 21 years, absence of any intrathoracic foreign material that might give a suspicion of the main pathology (e.g., port catheter might indicate the presence of lung cancer or potential pulmonary metastasis, thoracic tube might indicate pneumothorax history, etc.), posterior-anterior projection (PA) in standing position. Data were preselected by a radiology resident (three years of experience in thoracic imaging) in order to obtain a balanced dataset including four different pathologies (pneumonia, pleural effusion, pneumothorax, and pulmonary nodules) and also a subset of normal CXR without any pathological finding. Prevalences might be slightly higher than usually expected in the emergency unit to allow for a sufficient statistical analysis also with respect to usually low-frequent pathologies (e.g., pneumothorax, pulmonary nodules). Several of the initially identified images have been excluded with respect to inclusion criteria and trying to match a representative age- and gender-adapted collective (Figure 1A). In doing so, a series of 563 PA CXRs was collected (Figure 1B). The underlying DICOM files were exported anonymized from any personal data and handed over for reading purposes to nine different physicians working at the local university hospital. Six of the readers were physicians in the university hospital’s radiology department—three board-certified radiologists (BCR, 17 years of experience [YOE] in CXR reading, 9 YOE, 7 YOE) and three radiology residents (RR, 4 YOE, 3 YOE, 2 YOE). Furthermore, three additional readers were included, all of whom clinicians involved in the emergency department (non-radiology residents; NRR): one cardiology resident (4 YOE in ER), one gastroenterology resident (3 YOE in ER) and one traumatology resident (1 YOE in ER). It should be noted that the selection of readers did not include long-time trained emergency department specialists. They were excluded because they typically do not receive a specific CXR degree, making subgroup comparison difficult. Emphasis was placed on comparing RR and NRR readers because these are usually the first physicians to perform CXR interpretation in ED. BCR readers, who are usually responsible for confirming or denying written diagnostic reports, served as the control group (gold standard). All readers had to annotate the cases side-separately for the probability of a suspected pathology (pneumonia, pleural effusion, pneumothorax, pulmonary nodule). In addition, co-occurence of pathologies would be possible. Probability was determined on a Likert scale from 0 to 4 (0—no suspicion of pathology, 1—unlikely, 2—possible, 3—likely, 4—safe presence) [15] twice per case, one for each hemithorax (right and left). In the case of detected nodules, readers had to additionally note if they consider malignancy and would therefore recommend a follow-up computed tomography (CT) scan. All readers received thorough verbal and uniform written instructions prior to the reading process. The radiology resident who preselected the study cohort (Figure 1A) did not take part in the main reading.

### 2.2. Statistics

All statistical calculations as well as graphic illustrations have been performed using open-source programming language R [16]. Due to the presence of ordinal data (Likert scale), mainly non-parametric tests were used.

Consensus was built by summing up the individual readers’ confidence scores within the specified medical expert groups: BCR, RR, and NRR. Likert-scale decision analysis was performed using Kruskal–Wallis one-way analysis of variance with the addition of post hoc Dunn’s test of multiple comparisons with Šidák correction. Interrater reliability (>2 reader, >2 consensus) was calculated with Kendall’s coefficient of concordance (Kendall’s W). Groupwise correlation (*n* = 2) was performed with Spearman’s Rho. Results were considered significant if *p* < 0.05.

## 3. Results

### 3.1. Reading Duration

Reading duration was measured individually by the readers (not objectively). Overall reading duration was 6.5, 7.0 and 15 h in group BCR, 5.0, 9.0 and 9.0 h in group RR and 16.8, 17.0 and 20.0 h in group NRR. This results in a mean reading duration of 10.2 h for group BCR, 7.7 h for group RR and 17.9 h for group NRR.

### 3.2. Distribution of Likert Scale-Based Diagnosis

**Figure 2:**
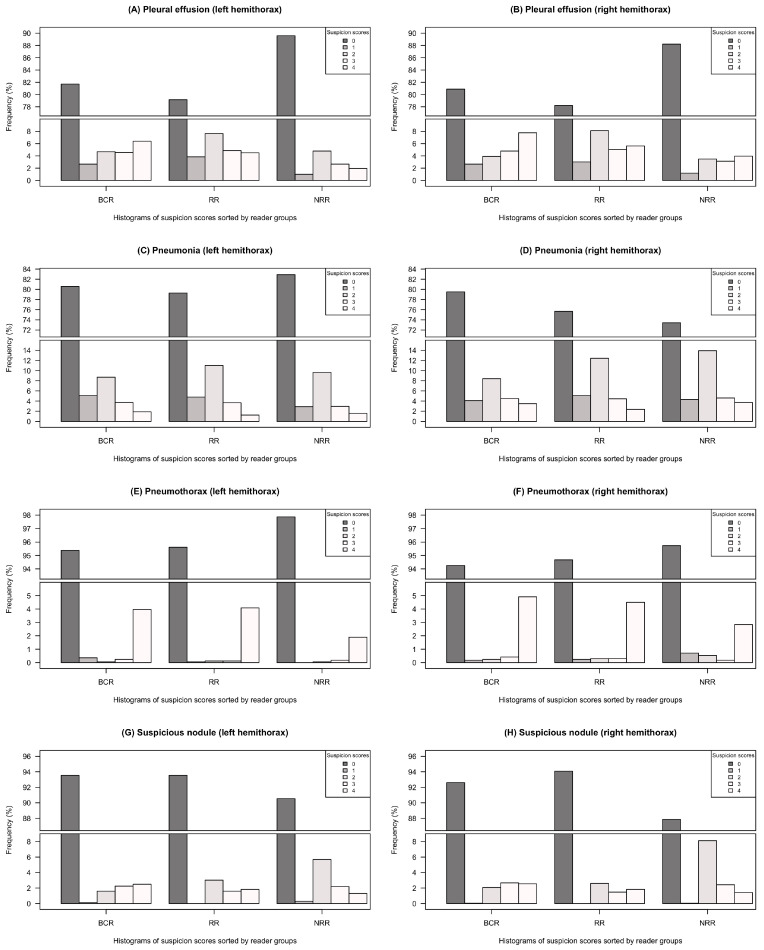
Distribution of Likert-scale based choices (0–4) separated by groups BCR (board-certified radiologists), RR (radiology residents) and NRR (non-radiology residents—Graphs contain gaps in *y*-axes since option 0 (no suspicion of pathology) was chosen most frequently in all pathologies and groups (**A**–**H**). Frequency is given in % of all individual answers in the reading group. Individual choice distribution can be found in the Supplement (Figure S1).

Figure 2 graphically summarizes the distribution of choices among the three groups of readers (BCR, RR and NRR) based on the given Likert scale (0–4). Distribution of the individual reading choices can be found in the Supplement (Figure S1). Table 1 presents the statistical analysis of differences in group consensus comparison. The consideration of all BCR choices of options > 1 as a positive pathology will result in higher overall pathology frequencies than preselected by the radiology resident (as shown in Figure 1) due to this very sensitive BCR reading interpretation. Pleural effusion was more often diagnosed within the groups of BCR and RR than in NRR—differences were statistically significant for both hemithoraces (*p* < 0.001 in all cases), see Figure 2A,B/Table 1. All three groups (BCR, RR, NRR) chose indifferent options 1–3 for pleural effusion assessment with a high frequency. Similarly, in terms of interpretation uncertainty, all groups would most often choose the indifferent option 2 if they suspected any presence of pneumonia, see Figure 2C,D. While suspicion of pneumonia in the left hemithorax was quite similar between the reader groups, RR and NRR suspected pneumonia in the right hemithorax more often than BCR, with statistically significant differences in comparisons of BCR/RR to NRR (BCR–NRR: *p* < 0.001, RR–NRR: *p* = 0.007, BCR–RR: *p* = 0.789 ), see Figure 2C,D/Table 1. In contrast to pleural effusion/pneumonia detection, pneumothorax was basically assessed as a yes-or-no-call—all groups mainly decided between options 0 or 4, whereas intermediate options 1–3 were chosen less frequently, see Figure 2E,F. No statistically significant group-related differences could be observed for pneumothorax detection, see Table 1. In terms of suspicious nodules, huge discrepancies could be observed in between groups of BCR/RR and group NRR (*p* < 0.001 in every case, except RR–NRR [left hemithorax]: *p* = 0.001), see Table 1. In terms of interpretation uncertainty, NRR was more likely to choose the indifferent option 2 for nodule detection, see Figure 2G,H.

### 3.3. Interrater Reliability

Table 2 side-separately highlights the results of interrater comparisons which were quantified by inter-individual agreements (readers considered individually) as well as by consensus agreements (comparing the consensus of different reader groups). Overall agreement showed differences according to pathologies and thorax sides (left [LH] and right hemithorax [RH]). Highest overall agreement values were reached in the pathology pneumothorax (overall-inter-individual agreement: $W_{LH}=0.719$, $W_{RH}=0.710$; overall-consensus agreement: $W_{LH}=0.806$, $W_{RH}=0.747$). Lowest overall agreement values were found in the detection of suspicious nodules (overall-inter-individual agreement: $W_{LH}=0.391$, $W_{RH}=0.417$; overall-consensus agreement: $W_{LH}=0.578$, $W_{RH}=0.595$). Detection of pleural effusion (overall-inter-individual agreement: $W_{LH}=0.562$, $W_{RH}=0.647$; overall-consensus agreement: $W_{LH}=0.787$, $W_{RH}=0.812$) showed higher overall agreement values than detection of pneumonia (overall-inter-individual agreement: $W_{LH}=0.532$, $W_{RH}=0.568$; overall-consensus agreement: $W_{LH}=0.732$, $W_{RH}=0.760$).

Considerable side differences could be observed for every pathology: With exception of the detection of pneumothorax, all pathologies showed better results in overall-inter-individual agreement and overall-consensus agreement for pathologies in the right hemithorax, whereas values on the left side were usually lower. Consensus agreement was highest in the comparison BCR–RR (BCR/RR-consensus agreement; highest to lowest agreement values were: pleural effusion > pneumothorax > pneumonia > suspicious nodule). Comparisons BCR–NRR (BCR/NRR-consensus agreement) and RR–NRR (RR/NRR-consensus agreement) showed lower agreement values for all pathologies (highest to lowest agreement values were: pneumothorax > pleural effusion > pneumonia > suspicious nodule). Very poor agreement was found in the detection of suspicious nodules in the comparisons BCR–NRR (BCR/NRR-consensus agreement: $\rho_{LH}=0.300$, $\rho_{RH}=0.359$) and RR–NRR (RR/NRR-consensus agreement; $\rho_{LH}=0.303$, $\rho_{RH}=0.417$).

Agreement among the groups’ individual readers was highest in group RR (RR-inter-individual agreement)—directly followed by BCR (BCR-inter-individual agreement) and lowest in group NRR (NRR-inter-individual agreement) for almost all pathologies (except pneumothorax right hemithorax: BCR-inter-individual agreement > RR-inter-individual agreement).

### 3.4. Potentially Missed Findings

**Figure 3:**
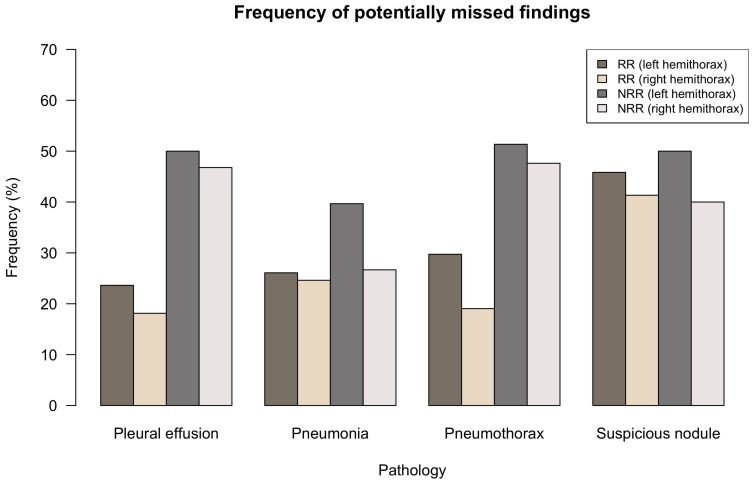
Potentially missed findings—Comparison of BCR consensus (serving as underlying reference standard) with RR and NRR. The graph shows the fraction of all cases in which the RR-/NRR-consensus were exactly 0 (consensus being defined as the sum of the groups’ three individual reading choices with a value range from 0–12) as a percentage of all cases with the BCR consensus exceeding 0. It therefore shows the fraction of cases that were potentially missed by all RR/NRR groups’ individual readers.

Figure 3 quantifies the fraction of cases with the RR and NRR consensus being exactly 0 as a percentage of all cases with BCR consensus (serving as reference standard) exceeding 0 for the considered pathology/hemithorax. This analysis sensitively quantifies how many cases of pathologies might have been overseen by RR/NRR consensus; it can therefore give an idea of how many findings were potentially missed by all readers in RR or NRR group but detected by at least one BCR. For all pathologies, potentially missed findings were higher in NRR group than in RR group, but differences were smaller in the detection of suspicious nodules. In the pathologies’ pleural effusion, the pneumonia and pneumothorax RR group had comparable frequencies of potentially missed findings of approx. 20–30%. Side-separated evaluation shows a surplus of missed findings in the left hemithorax.

## 4. Discussion

The present study employed a quantitative approach to investigate diagnostic agreement of differently qualified medical experts in the interpretation of emergency unit chest radiographs. We demonstrated that interpretation of PA CXRs can show major discrepancies depending on both the pathology (to detect in a side-dependent fashion) and the medical experts’ qualification. To our knowledge, this is the first reading that statistically focuses on CXR interpretation uncertainty in a representative emergency unit setting and includes radiologists as well as non-radiologists.

**Figure 4:**
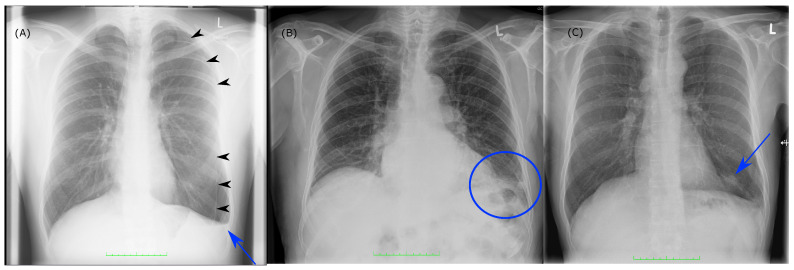
Example cases of the study—(**A**) patient with the finding of a seropneumothorax, the pleural dehiscence is marked with black arrowheads (detected by BCR, RR, but missed by 2/3 of NRR, the one NRR reader who found the dehiscence though picked the indifferent option 2 on the 0–4 Likert scale), the pleural effusion is marked with a blue arrow (detected by 2/3 BCR and 3/3 RR, missed by 3/3 NRR); (**B**) patient with a consolidation in the left basal lung. 3/3 BCR and 2/3 RR claimed that pneumonia might be possible (option 2). 0/2 NRR suspected pneumonia. Pleural effusion was suspected by 1/3 BCR, 2/3 RR, 2/3 NRR (options 2 and 3); (**C**) patient with a nodule in the basal left lung (blue arrow) which was detected and classified as potentially suspicious by 3/3 BCR (always option 4), 3/3 RR (options 4/2/2) and only 1/3 NRR (options 3/0/0).

Best overall-inter-individual agreement was shown for pneumothorax detection. As detection of pneumothorax might require immediate treatment, it is without doubt one of the most important pathologies for ED physicians and therefore needs to be time-critically detected. Results yielded that the detection was mainly a yes-or-no-call, since the intermediate suspicion scores (1–3) were disproportionally underrepresented in all groups (Figure 2E/F). We could also see that the number of potentially missed findings was very high in group NRR with values up to over 50% (Figure 3). Figure 4A correspondingly illustrates an example in which even clear and relevant findings were missed by most non-radiologists. Considering the pathologies’ pleural effusion and pneumonia, we could observe a predominance of insecure suspicion scores 1–3 in all groups (Figure 2A–D) and a lower overall-inter-individual agreement than for pneumothorax detection. However, this could be improved by considering the overall-consensus agreement (Table 2). The better consensus agreements might be explained by the fact that the consensus as defined by the sum of the individual reading choices gets comparable between the groups if individual readers mainly decide for indifferent options (1–3) and if statistical outliers are getting less important. Furthermore, we could note statistically significant differences for both pathologies (pleural effusion and pneumonia) by comparing BCR–RR and BCR–NRR (except pathology pneumonia in left hemithorax, Table 1): Comparing the radiologist’s groups (BCR–RR), on the contrary, no statistically significant differences were found. In addition, the frequency of potentially missed findings was higher in group NRR than in RR (Figure 3). We can therefore assume that non-radiologists had more difficulties in the detection of pleural effusion and pneumonia than radiology residents considering the board-certified radiologists’ suspicion scores as a reference standard. In the pathology pleural effusion, we furthermore noted that radiologists tend to express suspicion more often than non-radiologists since BCR and RR groups chose option 0 less frequently than group NRR (Figure 2A,B). A more sensitive pleural effusion detection rate can be of clinical advantage as even a small pleural effusion might have to be controlled or even treated—uncertainty in pleural effusion detection can also be easily and quickly validated by an additional ultrasound of the pleura [17]. Example case (B) in Figure 4 shows that a certain overlap might have occurred in the detection of consolidation suspicious of pneumonia and pleural effusion when pathologies were found in the basal lungs.

The lowest overall agreement values were found in the detection of suspicious nodules. Especially overall-inter-individual agreement was very low ($W_{LH}$ = 0.391, $W_{RH}$ = 0.417, Table 2). Considering the distribution of suspicion scores, it is striking that non-radiologists more frequently chose the indifferent option 2 than did the radiologists (Figure 2G/H). In addition, agreement among the three individual NRR readers was lower than in the other intragroup comparisons (Table 2). This implies that NRR had many insecurities in the detection of potentially malignant pulmonary nodules which can also be seen in example case (C) of Figure 4.

Results further showed side differences comparing the left and right hemithorax. In all pathologies (except pneumothorax), interrater reliability coefficients were higher and potentially missed findings lower in the right hemithorax. We infer that the cardiac projection is the cause for this observation as it covers a huge part of the left hemithorax in a PA CXR. The only exception from this phenomenon could be observed whilst analyzing the pathology pneumothorax (Table 2). Since most pleural dehiscences are located in the upper or lateral thoracic region, this detection area usually does not interfere with the cardiac projection.

In all pathologies, the lowest inter-individual agreement was noticed within the NRR group (Table 2). While in pathologies like pleural effusion (left-sided) pneumonia and pneumothorax detection rates were lower than in radiologists’ groups, suspicious nodules were more frequently detected by NRR and insecurities were higher in NRR than in BCR/RR (Figure 2). Moreover, potentially missed findings were higher in an NRR group than in an RR group for the pathologies pleural effusion, pneumonia and pneumothorax (Figure 3), a fact that can be of acute importance, especially in an ED setting without a 24/7 radiology department present. The results are consistent with results obtained by Eisen et al., which compared reading competence of radiology residents to that of readers working in intensive care and internal medicine departments and also to that of medical students [14]. When comparing experience and reading durations among RR and NRR, we observed that whilst RR and NRR have comparable experience time (RR: mean 3.0 YOE, NRR: mean 2.7 YOE), overall reading duration was significantly higher in NRR (RR: mean 7.7 h, NRR: mean 17.9 h, *p* = 0.004 in a Student’s *t*-test). We therefore might infer that NRR in ED profit from radiology reports in terms of both time efficiency and quality of reports. This might be of great importance in a setting without 24/7 coverage of a radiology department, which is often the case in smaller hospitals. In this scenario, non-radiology residents are usually the first CXR interpreters and have to make initial therapy decisions often based on their image analysis. In recent years, a number of artificial intelligence (AI) solutions have been released that aim to mimic the diagnostic performance level of medical specialists when interpreting radiographs, some of them showing promising results [18,19,20,21,22,23,24]. However, there have also been studies that revealed potential confounders in algorithm training which would lead to altered performance rates when applying the algorithm to different cohorts [25,26,27]. In a follow-up study to the one presented, we have applied a CXR detecting AI algorithm to the presented cohort showing a solid AI performance [28]. Future potential AI applications in the emergency department are discussed in detail there.

To our knowledge, the current study is the first reading study that evaluates CXR reading performance in the emergency department. With a large number of evaluated images (563 CXRs), and a high number of different readers (nine readers) with different levels of expertise, it can give a good overview about interpretation discrepancies that take place in the ED setting. Evaluation was proven on four very relevant and commonly diagnosed pathologies. Considering BCR’s reading results as a gold standard, the study offers a high qualified selection of readers with one BCR having an experience in CXR interpretation of 17 years. However, the study also has a number of limitations: Evaluation of findings is limited to the determined four pathologies. Long-time trained ED experts who are not in the radiology department but have been working in a clinical subdivision of the ED for several years were not involved in the reading process. Selection of cases was performed by a radiology resident and not randomly, which might have led to a small selection bias. Diagnoses were not validated by other diagnostics (blood tests, CT scans, etc.). Only CXRs in upright position (PA projection) were considered, leaving out lateral projection and supine projections which are also commonly acquired in ED. A certain bias might additionally result from the fact that RR were trained by BCR, which makes agreements between these two groups more likely.

## 5. Conclusions

Our study shows that major discrepancies in the detection of relevant CXR pathologies mainly occur by comparing radiologists’ and ED-experienced non-radiologists’ reading results. Especially in a setting lacking a 24/7 coverage by a radiology department or long turn-around times of radiology reporting this effect might be of great importance.

## Figures and Tables

**Table 1:** Test results showing statistically significant differences in consensus—Tests were performed using Kruskal–Wallis one-way analysis of variance and post hoc Dunn-tests with adjusted *p*-Values calculated by Šidák correction. Statistically significant results (*p* < 0.05) were illustrated in bold print. (*) No further post hoc test was perfomed because Kruskal–Wallis test was not statistically significant.

	Left Hemithorax (LH)	Right Hemithorax (RH)
Pleural effusion		
Kruskal–Wallis	$\chi^{2}=34.9$, df=2, ***p***** < 0.001**	$\chi^{2}=30.5$, df=2,***p***** < 0.001**
BCR–RR	p = 0.968	*p* = 0.898
BCR–NRR	***p*** **< 0.001**	***p*** **< 0.001**
RR–NRR	***p*** **< 0.001**	***p*** **< 0.001**
Pneumonia		
Kruskal–Wallis	$\chi^{2}=0.2$, df=2, *p* = 0.894	$\chi^{2}=16.6$, df=2,***p***** < 0.001**
BCR–RR	(*)	*p* = 0.789
BCR–NRR	(*)	***p*** **< 0.001**
RR–NRR	(*)	***p*** **= 0.007**
Pneumothorax		
Kruskal–Wallis	$\chi^{2}=5.4$, df=2, *p* = 0.066	$\chi^{2}=0.0$, df=2,*p* = 0.986
BCR–RR	(*)	(*)
BCR–NRR	(*)	(*)
RR–NRR	(*)	(*)
Suspicious nodule		
Kruskal–Wallis	$\chi^{2}=21.0$, df=2, ***p*****< 0.001**	$\chi^{2}=46.3$, df=2,***p***** < 0.001**
BCR–RR	*p* = 0.854	*p* = 0.796
BCR–NRR	***p*** **= 0.001**	***p*** **< 0.001**
RR–NRR	***p*** **< 0.001**	***p*** **< 0.001**

**Table 2:** Quantification of interrater and consensus agreements by interrater reliability and correlation analysis—Kendall’s coefficient of concordance (Kendall W) was calculated for overall-inter-individual agreement, inter-individual agreement among group’s readers (BCR, RR and NRR) and overall-consensus agreement. Consensus agreement comparing the three reading groups (BCR, RR and NRR) pairwise was established with interrater correlation (Spearman’s Rho). Different tests were performed because the number (*n*) of compared reading results differed (in consensus agreement *n* = 2, while *n* = 3 in BCR/RR/NRR-inter-individual agreement and overall-consensus agreement´ and *n* = 9 in overall-inter-individual agreement). Spearman’s Rho was used if *n* = 2 and Kendall W if *n* > 2. All calculated values showed *p* < 0.001.

	Overall-Inter-	BCR/RR-	BCR/NRR-	RR/NRR-	Overall-	BCR-Inter-	RR-Inter-	NRR-Inter-
	Individual	Consensus	Consensus	Consensus	Consensus	Individual	Individual	Individual
	Agreement	Agreement	Agreement	Agreement	Agreement	Agreement	Agreement	Agreement
	(*n* = 9)				(*n* = 3)	(*n* = 3)	(*n* = 3)	(*n* = 3)
	**Kendall W**	**Spearman** ρ	**Spearman ρ**	**Spearman ρ**	**Kendall W**	**Kendall W**	**Kendall W**	**Kendall W**
Pleural effusion								
Left hemithorax (LH)	0.562	0.774	0.626	0.648	0.787	0.654	0.756	0.663
	(*p* < 0.001)	(*p* < 0.001)	(*p* < 0.001)	(*p* < 0.001)	(*p* < 0.001)	(*p* < 0.001)	(*p* < 0.001)	(*p* < 0.001)
Right hemithorax (RH)	0.647	0.799	0.671	0.693	0.812	0.742	0.772	0.750
	(*p* < 0.001)	(*p* < 0.001)	(*p* < 0.001)	(*p* < 0.001)	(*p* < 0.001)	(*p* < 0.001)	(*p* < 0.001)	(*p* < 0.001)
Pneumonia								
Left hemithorax (LH)	0.532	0.696	0.509	0.590	0.732	0.685	0.703	0.584
	(*p* < 0.001)	(*p* < 0.001)	(*p* < 0.001)	(*p* < 0.001)	(*p* < 0.001)	(*p* < 0.001)	(*p* < 0.001)	(*p* < 0.001)
Right hemithorax (RH)	0.568	0.709	0.550	0.669	0.760	0.676	0.763	0.623
	(*p* < 0.001)	(*p* < 0.001)	(*p* < 0.001)	(*p* < 0.001)	(*p* < 0.001)	(*p* < 0.001)	(*p* < 0.001)	(*p* < 0.001)
Pneumothorax								
Left hemithorax (LH)	0.719	0.773	0.665	0.725	0.806	0.827	0.898	0.718
	(*p* < 0.001)	(*p* < 0.001)	(*p* < 0.001)	(*p* < 0.001)	(*p* < 0.001)	(*p* < 0.001)	(*p* < 0.001)	(*p* < 0.001)
Right hemithorax (RH)	0.710	0.825	0.515	0.521	0.747	0.861	0.843	0.726
	(*p* < 0.001)	(*p* < 0.001)	(*p* < 0.001)	(*p* < 0.001)	(*p* < 0.001)	(*p* < 0.001)	(*p* < 0.001)	(*p* < 0.001)
Suspicious nodule								
Left hemithorax (LH)	0.391	0.561	0.300	0.303	0.578	0.607	0.679	0.502
	(*p* < 0.001)	(*p* < 0.001)	(*p* < 0.001)	(*p* < 0.001)	(*p* < 0.001)	(*p* < 0.001)	(*p* < 0.001)	(*p* < 0.001)
Right hemithorax (RH)	0.417	0.623	0.359	0.291	0.595	0.686	0.632	0.509
	(*p* < 0.001)	(*p* < 0.001)	(*p* < 0.001)	(*p* < 0.001)	(*p* < 0.001)	(*p* < 0.001)	(*p* < 0.001)	(*p* < 0.001)

## Data Availability

No public data were used. The presented cohort is also used in Rudolph et al. [28].

## Supplementary Materials

The following figure is available online at https://www.mdpi.com/article/10.3390/diagnostics11101868/s1, Figure S1: Quantity of Likert-scale based choices (0–4) for the individual reader of groups BCR (board-certified radiologists 1–3), RR (radiology residents 1–3) and NRR (non-radiology residents 1–3) and all pathologies (**A**–**H**). Graphs contain gaps in *y*-axes since choice 0 (no suspicion of pathology) was chosen most frequently in all pathologies and readers. Frequency is given in absolute quantities of choices for each pathology.

## Abbreviations

The following abbreviations are used in this manuscript:

AI	artificial intelligence
BCR	board-certified radiologist(s)
CT	computed tomography
CXR	chest radiography
CXRs	chest radiographs
ED	emergency department
IM	internal medicine
LH	left hemithorax
NRR	non-radiology resident(s)
PA	posterior anterior projection
PACS	picture archiving and communication system
RH	right hemithorax
RR	radiology resident(s)
YOE	years of experience
