# Supplementary figures and images for: Interpretation of Thoracic Radiography Shows Large Discrepancies Depending on the Qualification of the Physician—Quantitative Evaluation of Interobserver Agreement in a Representative Emergency Department Scenario
Source: Diagnostics (Basel). 2021 Oct 11;11(10):1868. doi: 10.3390/diagnostics11101868 (PMC8534346; doi:10.3390/diagnostics11101868)

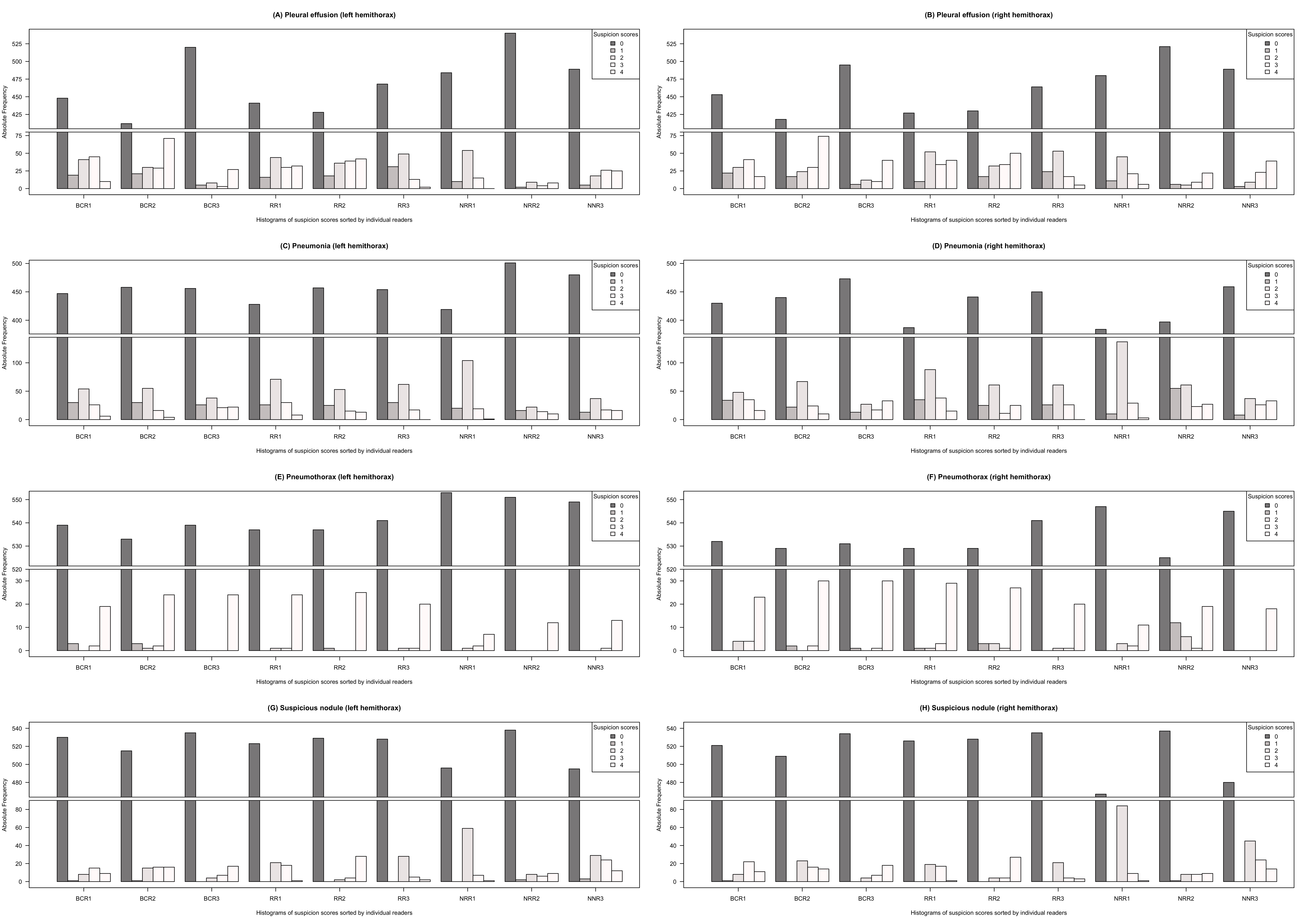

Supplement: Supplementary file 1 [file diagnostics-11-01868-s001.zip › diagnostics-1350799-supplementary.jpeg]
